# The ubiquitin system in heart and vessel pathobiology: From mechanisms to therapeutic strategies

**DOI:** 10.1016/j.gendis.2025.102023

**Published:** 2026-01-02

**Authors:** Jingjing Zhu, Zhimei Qiu, Yi Xu, Qing Guo, Shuangya Yang, Yongchao Zhao, Bei Shi

**Affiliations:** Department of Cardiology, Affiliated Hospital of Zunyi Medical University, Zunyi, Guizhou 563000, China

**Keywords:** Atherosclerosis, Cardiac remodeling, Deubiquitination, E3 ubiquitin ligase, Myocardial infarction, Post-translation modification, Ubiquitin proteasome system, Ubiquitination

## Abstract

Cardiovascular disease is currently a major global challenge, and its causes are complex, encompassing genetic, lifestyle, environmental, and other factors. Ubiquitination, an important post-translational protein modification, is closely associated with cardiovascular disease and is involved in the regulation of protein degradation, signaling, and gene expression. An increasing number of studies have shown that ubiquitination plays a key regulatory role in the development and progression of cardiovascular disease. Recent research has elucidated the crucial role of ubiquitination modifications in governing various cellular processes, signaling pathways, and protein homeostasis within cardiovascular contexts. Specifically, these modifications have been implicated in cardiomyocyte injury and hypertrophy, macrophage inflammation, phenotypic changes in smooth muscle cells, and activation of fibroblasts. This review summarizes the role of ubiquitination modifications in recent years, focusing on the recognition of different substrate proteins by E3 ubiquitin ligases. These ligases are involved in the regulation of various cardiovascular disorders, such as atherosclerosis, myocardial ischemia/reperfusion injury, cardiac remodeling, cardiac arrhythmia, and hypertension. The findings provide new insights into the prevention and treatment of cardiovascular disease.

## Introduction

### The biology of ubiquitination

**Figure 1:**
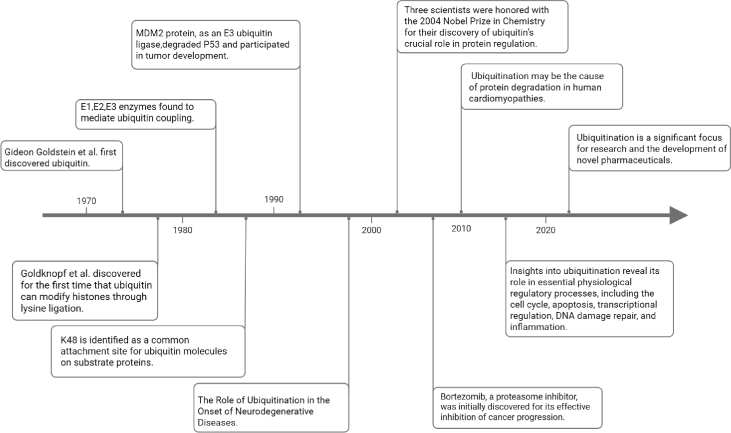
Timeline of the research process on ubiquitination.^7^, ^8^, ^9^, ^11^, ^12^, ^13^, ^14^, ^15^, ^16^, ^17^, ^18^, ^19^, ^20^, ^22^

Post-translational protein modifications regulate protein activity, localization, expression, and interactions with other cellular molecules through the covalent addition or removal of specific motifs on amino acid residues. Numerous post-translational modifications have been identified, and the list continues to expand, thereby greatly broadening the scope of proteomic research.^1^ Common examples include phosphorylation, acetylation, methylation, glycosylation, and ubiquitination.^2^ Ubiquitination is a well-established mechanism for endogenous protein degradation via the ubiquitin–proteasome system and is involved in almost all aspects of eukaryotic biology.^3^ This process is mediated through a multienzyme cascade that constitutes a central mechanism for regulating protein function.^4^ Ubiquitin, first discovered by Gideon Goldstein in 1975 (Fig. 1), is a 76–amino acid polypeptide that is highly conserved and widely expressed in eukaryotes. Structurally, ubiquitin consists of an amino-terminal, a carboxy-terminal, and a linear polypeptide chain containing seven lysine residues: K6, K11, K27, K29, K33, K48, and K63.^5^

**Figure 2:**
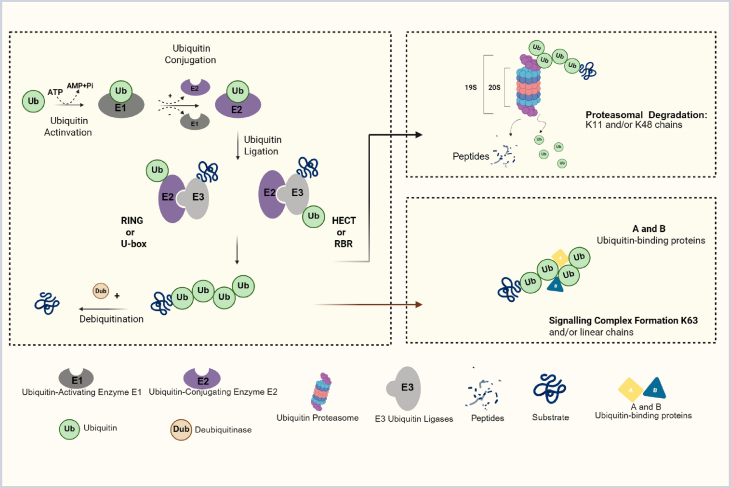
The regulatory process of ubiquitination. The ubiquitin molecule activates E1 in an ATP-consuming manner to form the E1-ubiquitin intermediate. Subsequently, the ubiquitin molecule transfers from E1 to the E2 enzyme to create the E2-ubiquitin intermediate. Finally, the E3 ligase binds to the target protein, leading to the formation of the ubiquitin-tagged target protein. Two types of E3 ligases are involved in this process: RING and U-box-type E3, which transfer to substrate proteins through an intermediate complex of E3 and E2-Ub, and RBR and HECT-type E3, where E2-Ub interacts to transfer ubiquitin to E3, which in turn transfers ubiquitin to the substrate. Ubiquitin chains linked by K48 and K11 facilitate the direct degradation of substrates in the proteasome, while mono-ubiquitinated and K63-linked ubiquitin chains serve non-protein hydrolytic functions. E1, ubiquitin-activating enzyme; E2, ubiquitin-conjuating enzyme; E3, ubiquitin ligases; Ub, ubiquitin; Dub, deubiquitinase.

In 1977, Goldknopf et al first demonstrated that ubiquitin can modify histones through lysine ligation.^6^ Each lysine residue of ubiquitin has the potential to undergo ubiquitination, which can occur as mono-ubiquitination (single ubiquitin attachment) or poly-ubiquitination (formation of ubiquitin chains). Polyubiquitin chains are typically linked via lysine residues such as K48 and K11 and serve as signals for proteasomal degradation, whereas mono-ubiquitination and K63-linked chains perform non-proteolytic functions.^23^, ^24^, ^25^ The ubiquitin–proteasome system involves three key enzymatic steps: activation of ubiquitin by the E1 ubiquitin-activating enzyme, conjugation by the E2 ubiquitin-conjugating enzyme, and covalent attachment of ubiquitin to the substrate protein by the E3 ubiquitin ligase.^26^ Specifically, E1 activates ubiquitin to form an E1–ubiquitin intermediate, which is then transferred to E2 to generate an E2–ubiquitin intermediate. Finally, E3 ligases facilitate the transfer of ubiquitin from E2 to a lysine residue on the target protein, forming a covalent linkage between the C-terminal glycine of ubiquitin and the substrate.^27^ Among these enzymes, E3 ligases are regarded as the most critical due to their substrate specificity and regulatory role in ubiquitination. More than 800 E3 ligases have been identified in the human genome, which can be broadly classified into four families based on structural domains: HECT, RING, U-box, and RBR.^28^ RING and U-box E3 ligases typically form an intermediate complex with E2–ubiquitin before substrate transfer, whereas HECT and RBR ligases first accept ubiquitin onto a catalytic cysteine before transferring it to the substrate. Iterative cycles of this process result in the formation of polyubiquitin chains, which are subsequently recognized by the 26S proteasome for degradation.^29^ The 26S proteasome is a barrel-shaped complex composed of two 19S regulatory particles flanking a 20S catalytic core (Fig. 2). The 19S regulatory subunits are responsible for recognizing and unfolding polyubiquitinated substrates, as well as disassembling ubiquitin chains. The 20S catalytic core then cleaves the substrate into short peptides for further processing.^30^ Importantly, ubiquitin conjugation is reversible. Deubiquitinating enzymes (DUBs) can remove ubiquitin modifications, thereby antagonizing ubiquitination and altering protein fate.^31^ Through this balance of ubiquitination and deubiquitination, protein activity and cellular signaling are dynamically controlled.

### Cardiovascular disease and ubiquitination

**Figure 3:**
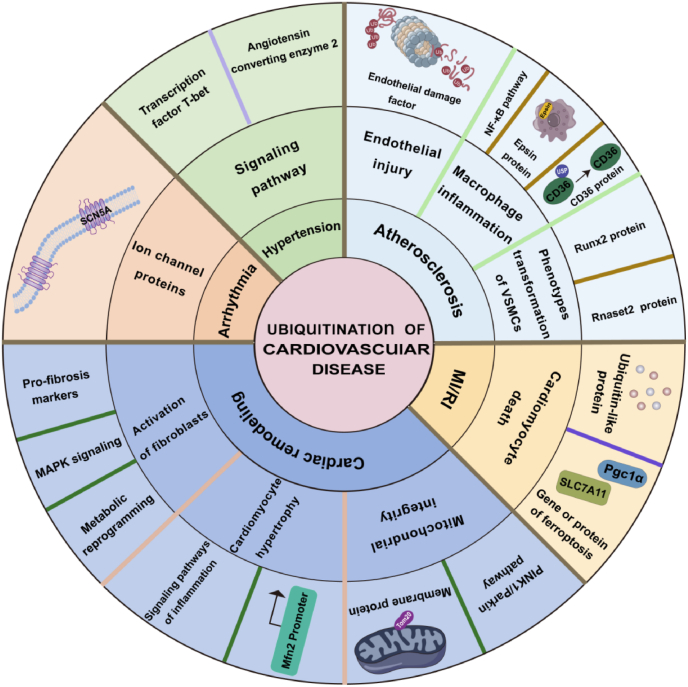
The role of ubiquitination in cardiovascular disease. The role of ubiquitination in cardiovascular disease spans across various conditions, including atherosclerosis, ischemia–reperfusion injury, cardiac remodeling, hypertension, and arrhythmias, regulating different cellular functions and signaling in different diseases. MI/R, myocardial ischemia–reperfusion. Created with Adobe Illustrator.

Cardiovascular disease encompasses a broad spectrum of disorders affecting the heart and blood vessels, including coronary artery disease, atherosclerosis, heart failure, arrhythmias, cardiomyopathy, and hypertension. These conditions remain a leading cause of morbidity and mortality worldwide, with their prevalence continuing to rise despite advances in pharmacological therapies and surgical interventions. Given their multifactorial and complex pathogenesis, a deeper mechanistic understanding of cardiovascular disease is urgently required. Ubiquitination has been increasingly recognized as a key contributor to the pathophysiology of systemic diseases by regulating protein turnover, cellular signaling, and inflammatory responses. A well-known example is the rapid degradation of p53 mediated by the E3 ubiquitin ligase MDM2, which promotes ubiquitination and proteasomal degradation of p53.^10^^,^^32^ Aberrant regulation of this pathway is closely associated with tumorigenesis. Beyond oncology, ubiquitination is also implicated in the regulation of neurodegenerative, metabolic, autoimmune, and inflammatory diseases.^33^, ^34^, ^35^, ^36^, ^37^ In recent years, accumulating evidence has highlighted the pivotal role of the ubiquitin–proteasome system in cardiovascular disease. The ubiquitin–proteasome system orchestrates key processes underlying the onset and progression of atherosclerosis, myocardial ischemia/reperfusion injury, cardiomyopathy, myocardial hypertrophy, and heart failure (Fig. 3).^38^ These insights underscore ubiquitination as a fundamental mechanism in cardiovascular biology and motivate the exploration of E3 ligases, DUBs, and ubiquitin-specific proteases (USPs) as potential therapeutic targets.

### Regulatory role of ubiquitination modifications in the cardiovascular system

Ubiquitination modifications and atherosclerosis.

Ubiquitination regulates vascular endothelial injury.

**Figure 4:**
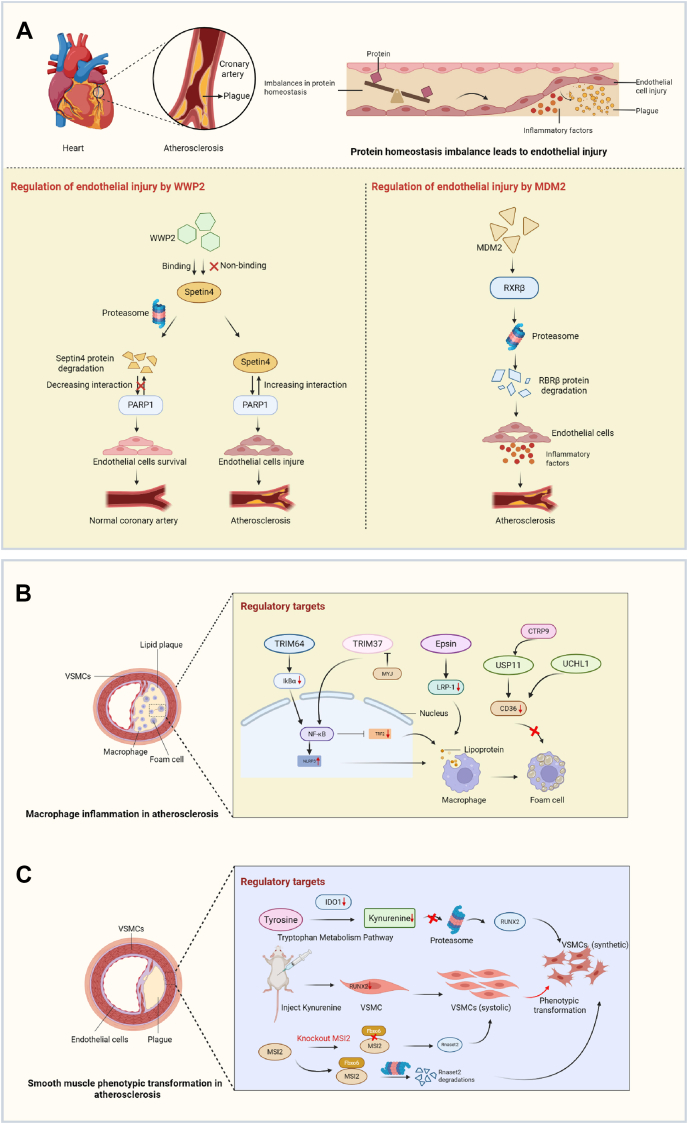
Regulatory targets of ubiquitination in atherosclerosis. Ubiquitination regulates homeostatic balance of proteins in endothelial cells **(A)**, macrophage inflammation **(B)**, and smooth muscle phenotypic **(C)**. WWP2, MDM2, TRIM64, TRIM37, and epsin are E3 ubiquitin ligases. USP11 and UCHL1 are deubiquitinases. VSMCs, vascular smooth muscle cells; Septin4, endothelial injury factor; RXRβ, retinoid X receptor-like β; MYJ, modified *Yuejuwan* (a traditional Chinese medicine); IDO1, indoleamine 2,3-dioxygenase 1; MSI2, Musashi-2 (a binding protein); LRP-1, low-density lipoprotein receptor-related protein 1; CTRP9, C1q/TNF-related protein 9.

Oxidative stress causes endothelial injury as an initial event and a major cause of atherosclerosis.^39^ A growing body of work indicates that loss of protein homeostasis within endothelial cells, rather than individual proteins alone, is a decisive determinant of endothelial dysfunction, and that HECT-type E3 ubiquitin ligases occupy a central position in this proteostatic network. For example, Zhang et al identified the GTPase Septin4 as a physiological substrate of the HECT E3 ligase WWP2 (Fig. 4). Mechanistically, WWP2 binds to Septin4 and catalyzes ubiquitination at K174, targeting Septin4 for proteasomal degradation; by limiting Septin4 accumulation and its interaction with PARP1, WWP2 reduces oxidative-stress-induced endothelial injury and downstream vascular remodeling.^40^ This study illustrates a recurring theme: endothelial fate can be determined by E3 enzymes that selectively remove pro-injury factors, thereby interrupting harmful protein–protein complexes. Complementary evidence points to an opposing strategy, in which pathological ubiquitination depletes protective regulators. Zeng et al showed that the E3 ligase MDM2 directly bound to and promoted ubiquitin-dependent degradation of RXRβ; pharmacologic inhibition of MDM2 in experimental systems lowered endothelial pro-inflammatory cytokine output and ameliorated atherosclerotic readouts, implicating MDM2 as a potential pro-atherogenic E3 in the endothelium.^41^ These examples demonstrate two mechanistic motifs in endothelial ubiquitin biology: i) selective clearance of pro-injury substrates (*e.g.*, Septin4) and ii) pathogenic removal of protective factors (*e.g.*, RXRβ). E3 ligases represent promising therapeutic targets, yet challenges remain in achieving substrate specificity and accounting for context-dependent effects of ubiquitination. Endothelium-focused ubiquitinome mapping and selective modulation strategies will be critical for translating these insights into therapy. In short, endothelial ubiquitination controls a balance between disposal of toxic effectors and preservation of protective regulators; understanding the E3–substrate networks, linkage specificity, and timing of intervention will be essential to translate these insights into safe, endothelium-targeted therapies.

### Ubiquitination regulates macrophage transformation and inflammation

Atherosclerosis is driven by chronic inflammation, in which macrophage transformation into foam cells plays a central role.^42^ Rather than a broad list of ubiquitin ligases, recent studies highlight several representative pathways that critically link ubiquitination to macrophage-driven inflammation.^43^ One example is TRIM64, a RING-type E3 ligase selectively induced by ox-LDL. TRIM64 directly ubiquitinates IκBα, leading to NF-κB activation and subsequent NLRP3 inflammasome assembly. This mechanism amplifies pro-inflammatory cytokine production and aggravates foam cell formation.^44^^,^^45^ These findings suggest that inhibiting TRIM64-mediated IκBα degradation may represent a promising strategy to dampen macrophage-driven inflammation. Another representative mechanism involves TRIM37, which is markedly up-regulated in foam cells. TRIM37 interacts with TRAF2 to promote NF-κB nuclear translocation, while its silencing enhances cholesterol efflux and reduces macrophage inflammatory responses. Interestingly, the traditional Chinese medicine modified *Yuejuwan* alleviates atherosclerosis by targeting the TRIM37/TRAF2/NF-κB axis,^46^ highlighting potential opportunities for pharmacological intervention. DUBs such as USP11 critically regulate lipid uptake in macrophages. USP11 stabilizes CD36 by preventing its ubiquitin-mediated degradation.^47^ Inhibition of USP11, or alternatively promoting CD36 ubiquitination through UCHL1 deletion,^48^ effectively reduces foam cell formation and lipid accumulation. This USP11/CD36 axis provides a clear example of how fine-tuning ubiquitination dynamics could limit macrophage lipid overload and attenuate atherosclerosis progression. Together, these representative examples underscore the central role of ubiquitination in controlling macrophage inflammation and foam cell formation. From a therapeutic perspective, targeting specific E3 ligases such as TRIM64^44^ or TRIM37,^46^ or modulating DUB activity in the USP11/CD36 pathway,^47^^,^^48^ offers translational potential. However, challenges remain in achieving substrate specificity, avoiding systemic immune suppression, and developing inhibitors with clinical feasibility. Future studies should therefore prioritize mechanism-based drug discovery to harness the ubiquitin system for atherosclerosis treatment.

### Ubiquitination regulates vascular smooth muscle (VSMC) phenotypic transformation

Atherosclerotic plaques range from cellular, collagen-rich (relatively stable) lesions to lipid-laden, vulnerable (unstable) plaques prone to rupture; VSMCs are central to this spectrum because their phenotypic plasticity can either stabilize lesions (matrix production, calcification) or promote instability (necrotic core formation, inflammation).^49^^,^^50^ Recent work has mechanistically linked ubiquitin-dependent control of lineage-specifying factors to VSMC reprogramming. For example, the tryptophan–kynurenine pathway (via indoleamine 2,3-dioxygenase 1, IDO1) restrains VSMC osteogenic conversion by promoting ubiquitin-dependent degradation of the osteogenic transcription factor, Runt-related transcription factor 2 (RUNX2). Exogenous kynurenine reduced arterial calcification and down-regulated RUNX2 in VSMCs, whereas IDO1 deficiency impaired proteasomal turnover of RUNX2; mechanistic evidence implicates an aryl-hydrocarbon-receptor (AhR)–dependent, non-genomic route that facilitates RUNX2 ubiquitination and clearance.^51^ This axis links metabolic cues to transcriptional control through targeted proteostasis, identifying nodes (IDO1 activity, AhR engagement, RUNX2 ubiquitination) that could be pharmacologically modulated to limit calcific remodeling. Complementary studies implicate RNA-binding proteins and E3/DUB networks in VSMC fate decisions. Zhang et al reported that Musashi-2 (MSI2), via interaction with the F-box protein Fbxo6, influenced VSMC phenotype and plaque development in murine atherosclerosis; MSI2 modulation altered RNaseT2 ubiquitination and downstream chemokine signaling, thereby affecting necrotic core size and lesion burden.^52^ Mechanistically, ubiquitin signaling shapes VSMC fate in two principal ways: by regulating the stability of lineage determinants (*e.g.*, RUNX2, RNA-binding proteins) and by integrating metabolic or inflammatory cues that convert reversible modifications into durable phenotypic switches. Therapeutically, pathways such as IDO1–AhR–RUNX2 and MSI2–Fbxo6–RNaseT2 represent promising targets, but translation is limited by substrate pleiotropy, systemic side effects, and redundancy within ubiquitin networks. Strategies that enable VSMC-selective or substrate-biased modulation hold greater promise. In summary, ubiquitin-mediated control of VSMC plasticity governs calcification and plaque stability. Future efforts should focus on validating these mechanisms in human tissues and developing targeted approaches that minimize systemic impact.

### Ubiquitination regulates cardiomyocyte death

Cardiomyocyte death represents a central event in myocardial ischemia–reperfusion injury, where distinct forms of cell loss determine functional recovery. Recent evidence highlights ubiquitination not merely as a degradative process, but as a regulatory hub coordinating multiple death pathways.^53^ In apoptotic signaling, the muscle-specific E3 ligase MuRF1 provides a well-studied example. By ubiquitinating contractile and stress-responsive proteins, MuRF1 limits ischemia-induced apoptosis and preserves myocardial structure.^54^ Notably, loss of MuRF1 exacerbates pathological hypertrophy and dysfunction,^55^ emphasizing its dual protective and regulatory role. These findings suggest that maintaining MuRF1 activity at an optimal threshold is critical, as both excessive inhibition and overactivation can be detrimental. Beyond apoptosis, ubiquitination also governs inflammatory crosstalk. The deubiquitinase USP11 stabilizes TRAF3, thereby sustaining NF-κB signaling and amplifying cardiomyocyte injury during reperfusion.^56^ Suppressing USP11 alleviates both inflammatory cytokine release and cell death, indicating that post-translational control of adaptor proteins can decisively shape cardiac outcome. However, targeting USP11 clinically requires caution, as broad suppression of NF-κB signaling might compromise host defense. In conclusion, ubiquitination plays a pivotal role in regulating cardiomyocyte apoptosis and inflammation-driven cell death during ischemia–reperfusion injury. Representative mechanisms, such as MuRF1-mediated protection against stress-induced apoptosis and USP11-dependent stabilization of TRAF3 that amplifies NF-κB signaling, illustrate how ubiquitin ligases and deubiquitinases can critically shape cardiac outcomes. These findings underscore the dual nature of ubiquitination, functioning as both a safeguard and a driver of injury depending on the molecular context. From a therapeutic perspective, precise modulation of these enzymes holds promise for limiting cardiomyocyte loss; however, the lack of substrate specificity and the risk of disrupting essential proteostasis remain major challenges that future studies must address. In conclusion, ubiquitination plays a pivotal role in regulating cardiomyocyte apoptosis and inflammation-driven cell death during ischemia–reperfusion injury. Representative mechanisms, such as MuRF1-mediated protection against stress-induced apoptosis and USP11-dependent stabilization of TRAF3 that amplifies NF-κB signaling, illustrate how ubiquitin ligases and deubiquitinases can critically shape cardiac outcomes. These findings underscore the dual nature of ubiquitination, functioning as both a safeguard and a driver of injury depending on the molecular context. From a therapeutic perspective, precise modulation of these enzymes holds promise for limiting cardiomyocyte loss; however, the lack of substrate specificity and the risk of disrupting essential proteostasis remain major challenges that future studies must address.

### Ubiquitination regulates ferroptosis in cardiomyocytes

**Figure 5:**
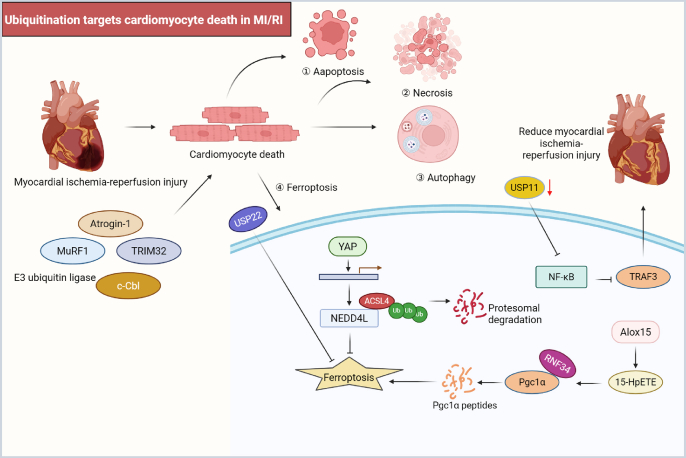
Regulatory targets of ubiquitination in MI/RI. In cardiomyocytes, cell death can occur through processes such as autophagy, necrosis, apoptosis, and ferroptosis. The E3 ubiquitin ligase has been identified as a key player in promoting cardiomyocyte death. Additionally, deubiquitinating enzymes are involved in mediating ferroptosis in cardiomyocytes through various signaling pathways. MI/RI, myocardial ischemia–reperfusion injury; Atrogin-1, MuRF1, TRIM32, and NEDD4L are E3 ubiquitin ligases; USP22 and USP11 are deubiquitinating enzymes. c-Cbl, the proto-oncogene Casita b-lineage lymphoma; YAP, yes-associated protein; Alox15, 15-lipoxygenase; RNF34, ring finger protein 34; 15-HpETE, 15-hydroperoxyeicosatetraenoic acid; ACSL4, acyl-CoA synthetase long-chain family member 4; Pgc1α, proliferator-activated receptor gamma coactivator 1-alpha.

Cardiomyocyte death can occur through various mechanisms, such as necrosis, apoptosis, autophagy, and ferroptosis. Consequently, safeguarding cardiac function after ischemia–reperfusion injury necessitates the prevention of cell death.^57^ Ferroptosis, an iron-dependent form of regulated cell death, has emerged as a crucial determinant of myocardial ischemia–reperfusion injury. Recent evidence indicates that ubiquitination does not merely participate in ferroptotic signaling but actively governs its initiation and resolution.^58^ A representative pathway involves the transcriptional co-activator YAP, which up-regulates the E3 ligase NEDD4L. NEDD4L targets ACSL4, a lipid-metabolizing enzyme indispensable for ferroptosis, for ubiquitin-mediated degradation. This regulatory axis suppresses ferroptotic damage and preserves cardiac function following reperfusion.^59^ In parallel, lipid peroxidation driven by Alox15 produces 15-HpETE, which promotes Pgc1α degradation through ubiquitin-dependent mechanisms. Pharmacological inhibition of Alox15 prevents ferroptosis and restores mitochondrial integrity, highlighting its translational potential.^60^ Additional modulators have also been identified. For instance, the deubiquitinase USP22 protects cardiomyocytes by stabilizing SIRT1/p53/SLC7A11 signaling and attenuating iron-driven oxidative stress.^61^ Collectively, these findings reveal that ubiquitination acts at multiple levels—ranging from lipid metabolism to redox regulation—to fine-tune ferroptotic susceptibility in cardiomyocytes. In summary, ubiquitination functions as a central checkpoint of cardiomyocyte ferroptosis. Targeting specific ligases or deubiquitinases, such as the YAP–NEDD4L–ACSL4 axis or USP22-mediated antioxidant defense, offers promising strategies to mitigate ischemia–reperfusion injury (Fig. 5). Nevertheless, the pleiotropic effects of ubiquitination and the challenge of achieving substrate selectivity underscore the need for precise, mechanism-based therapeutic interventions.

### Ubiquitination regulates cardiomyocyte hypertrophy

Pathological cardiac hypertrophy is a hallmark of maladaptive remodeling in cardiovascular disease and strongly predisposes to heart failure. Ubiquitination has emerged as a critical regulator of this process, functioning not only in protein turnover but also in the fine-tuning of hypertrophic signaling cascades.^62^ Among the best-studied examples, the muscle-specific E3 ligase MuRF1 plays a pivotal protective role. In mouse models of pressure overload or transverse aortic constriction, MuRF1 deficiency results in exaggerated hypertrophy and reduced left ventricular function. Mechanistically, MuRF1 directly ubiquitinates calcineurin A (CnA), decreasing its protein stability and thereby limiting NFAT-driven transcription of hypertrophic genes.^55^^,^^63^ This dual role—safeguarding against pathological hypertrophy but being potentially harmful when excessively suppressed—underscores the need for balanced MuRF1 activity. Other representative ligases exert distinct effects. RNF207 aggravates cardiac hypertrophy by ubiquitinating TAB1 and activating p38/JNK1/2 signaling in both pressure overload-induced mice and phenylephrine-stimulated cardiomyocytes.^64^ In contrast, RNF13 interacts with p62 to enhance NRF2/HO-1 signaling, conferring protection against oxidative stress–induced hypertrophy.^65^ Members of the TRIM family also exhibit context-dependent roles: TRIM32 reduces dysbindin-mediated SRF activation and attenuates hypertrophy in neonatal rat cardiomyocytes, whereas TRIM24 enhances SRF signaling and promotes growth. Likewise, overexpression of TRIM14 exacerbates hypertrophy both *in vivo* and *in vitro* via AKT pathway activation.^66^^,^^67^ In addition, the HECT-type ligase ITCH degrades Dvl proteins through ubiquitination, thereby inhibiting Wnt/β-catenin signaling and restraining hypertrophy.^68^ Recent reports suggest that vascular abnormalities may precede cardiomyocyte hypertrophy. The E3 ligase MDM2 destabilizes HIF1α and HIF2α, leading to imbalanced angiogenesis and microvascular dysplasia, which in turn aggravates ventricular remodeling. Down-regulation of MDM2 restores HIF protein levels and prevents vascular dysfunction, highlighting a novel axis for early therapeutic intervention.^69^

**Figure 6:**
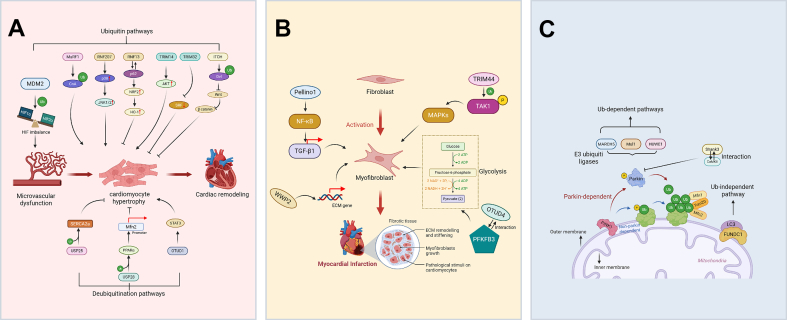
Regulatory targets of ubiquitination in cardiac remodeling. Ubiquitination plays a crucial role in cardiac remodeling by regulating various cellular functions and signaling pathways, such as cardiomyocyte hypertrophy **(A)**, fibroblast activation **(B)**, and mitochondrial function **(C)**. Tom20, Mfn1, and Mfn2 indicate the outer membrane of mitochondria. CnA, calmodulin A; JNK1/2, c-Jun N-terminal kinase; Ub, ubiquitin; HIF1α, hypoxia-inducible factor 1 alpha; HIF2α, hypoxia-inducible factor 2 alpha; SRF, serum-response factor; Mfn2, mitofusin2; PPARα, peroxisome proliferator-activated receptor α; TRIM, tripartite motif containing 44; TAK1, transforming growth factor-β-activated kinase 1; PFKFB3, phosphofructokinase-2/fructose-2,6-bisphosphatase 3; OTUD4, deubiquitinase OTU domain containing 4; Pellino 1, a member of the Pelle-interacting protein family; Parkin, parkin RBR E3 ubiquitin-protein ligase; Pink1, PTEN induced putative kinase 1; Shank3, SH3 and multiple ankyrin repeat domains protein 3; CaMKII, Ca^2+^/calmodulin-dependent protein kinase II; FUNDC1, FUN14 domain containing 1; LC3, light chain 3.

DUBs provide an additional layer of regulation. For example, USP25 stabilizes SERCA2a through deubiquitination, thereby improving calcium handling and attenuating hypertrophy and fibrosis.^70^ USP28 stabilizes PPARα, promoting Mfn2 transcription and protecting against diabetic cardiomyopathy.^71^ By contrast, OTUD1 enhances STAT3 activation through deubiquitination, driving inflammation, fibrosis, and hypertrophy, and thus contributes to heart failure progression.^72^ These findings illustrate that both ubiquitin ligases and DUBs critically shape hypertrophic signaling networks by controlling protein stability and transcriptional programs. In summary, cardiomyocyte hypertrophy is orchestrated by a limited set of ubiquitin-related enzymes, including MuRF1^55^^,^^63^, RNF207^64^, RNF13^65^, TRIM proteins^66^^,^^67^, ITCH^68^, MDM2^69^, and several DUBs such as USP25, USP28, and OTUD1.^70^, ^71^, ^72^ These examples illustrate the dual nature of ubiquitination: the same system can either restrain or exacerbate hypertrophic remodeling depending on the enzyme, substrate, and cellular context (Fig. 6A). From a therapeutic perspective, targeting these enzymes is promising but remains challenging. Key obstacles include achieving substrate and pathway specificity, minimizing off-target effects, and determining the optimal timing for intervention. Thus, while ubiquitination offers attractive opportunities for novel therapies, its pleiotropic functions demand careful and mechanism-based strategies for translation into clinical practice.

### Ubiquitination regulates fibroblast activation

Activation of cardiac fibroblasts is a central driver of maladaptive extracellular matrix remodeling and ventricular stiffening, ultimately culminating in heart failure. Recent studies implicate ubiquitin-dependent mechanisms as key regulators of fibroblast activation, converging on both canonical signaling cascades and emerging metabolic pathways. The E3 ligase Pellino1, up-regulated in pressure-overloaded rat fibroblasts, promotes pathological remodeling; its inhibition alleviates cardiac dysfunction, hypertrophy, and fibrosis.^73^ Similarly, WWP2 enhances the expression of profibrotic markers and extracellular matrix genes in primary fibroblasts.^74^ TRIM44 stabilizes TAK1 by blocking K48-linked ubiquitin-mediated degradation, thereby amplifying MAPK signaling and accelerating fibroblast activation.^75^ Beyond classical signaling, metabolic reprogramming has emerged as a novel dimension. Following myocardial infarction, TGF-β1-stimulated fibroblasts exhibit increased glycolysis through PFKFB3 up-regulation. The deubiquitinase OTUD4 stabilizes PFKFB3, sustaining glycolytic flux and fueling fibrotic remodeling.^76^ Together, these findings illustrate two converging paradigms: ubiquitination modulates canonical pro-fibrotic signaling (*e.g.*, TAK1/MAPK, TGF-β pathways) and intersects with metabolic reprogramming (*e.g.*, OTUD4–PFKFB3), thereby integrating stress and metabolic cues to drive fibroblast activation. Targeting fibroblast-specific ubiquitin enzymes holds promise for antifibrotic therapy. However, major obstacles remain: most identified ligases and DUBs (*e.g.*, Pellino1, TRIM44) are also involved in immune or tumor biology, raising concerns of systemic side effects; metabolic enzymes like PFKFB3 play dual roles in angiogenesis and repair, complicating selective targeting; and current evidence relies heavily on rodent models, with limited validation in human tissues. These issues underscore the need for cell type-specific delivery systems, better biomarkers for patient stratification, and mechanistic studies that capture the multicellular complexity of cardiac fibrosis (Fig. 6B).

### Ubiquitination regulates mitochondrial apoptosis and integrity

Heart failure is a leading cause of morbidity and mortality worldwide.^77^ Mitochondrial dysfunction has emerged as a critical pathogenic factor.^78^ Recent studies underscore the importance of mitochondrial quality control, particularly mitophagy and ubiquitin-dependent regulation, in preserving mitochondrial integrity and mitigating cardiac dysfunction.^79^ Among the most extensively studied pathways, the PINK1/Parkin axis plays a central role. Upon mitochondrial depolarization, PINK1 phosphorylates both Parkin and ubiquitin, thereby recruiting Parkin to the outer mitochondrial membrane. Parkin subsequently ubiquitinates proteins such as Tom20, Mfn1, and Mfn2, promoting mitophagy and maintaining mitochondrial integrity.^80^ Deficiency or dysfunction of Parkin results in impaired clearance of damaged mitochondria, which contributes to heart failure.^81^^,^^82^ In addition, MARCH5, Mul1, and HUWE1 are also implicated in mitochondrial regulation,^83^, ^84^, ^85^, ^86^, ^87^, ^88^ with aberrant activity leading to mitochondrial instability and cardiac remodeling. Recent findings suggest that other E3 ligases modulate mitochondria-dependent cell survival. For instance, TRIM65 suppresses mitochondria-dependent apoptosis and autophagy by regulating Jak1 ubiquitination, thereby attenuating isoproterenol-induced cardiac hypertrophy and improving cardiac function.^89^ Similarly, the mitochondrial outer membrane protein FUNDC1 serves as a receptor for hypoxia-induced mitophagy. Under hypoxic stress, dephosphorylated FUNDC1 interacts with LC3 to trigger selective mitophagy.^90^ However, MARCH5 antagonizes this process by ubiquitinating and degrading FUNDC1, thereby reducing mitochondrial sensitivity to hypoxia-induced mitophagy.^91^ Intriguingly, FUNDC1 also activates the ROS–HIF1α pathway, promoting pulmonary arterial smooth muscle cell proliferation and vascular remodeling, linking mitochondrial ubiquitination to pulmonary hypertension.^92^ Therapeutic modulation of mitochondrial ubiquitin signaling is gaining attention. Natural compounds such as berberine have been shown to enhance PINK1/Parkin-mediated mitophagy and protect against heart failure.^93^ Reticulin 1 improves infarction-induced heart failure by sustaining mitochondrial dynamics and activating SIRT3/FOXO3a-dependent mitophagy.^94^ Likewise, *NuanXinKang*, a Chinese medicinal preparation, up-regulates PINK1/Parkin-mediated mitophagy, preventing mitochondrial dysfunction and preserving cardiac function in ischemic heart failure.^21^ In summary, mitochondrial ubiquitination pathways—particularly the PINK1/Parkin axis, MARCH5- and FUNDC1-dependent mitophagy, and regulatory ligases such as TRIM65—play essential roles in safeguarding mitochondrial quality and shaping the course of heart failure (Fig. 6C). Yet, their functions are highly context-dependent: insufficient mitophagy accelerates the accumulation of dysfunctional mitochondria, whereas excessive activation may compromise cellular energetics. Moreover, distinct patterns of mitochondrial remodeling have been observed in different heart failure subtypes (*e.g.*, HFrEF *vs*. HFpEF), raising the possibility that ubiquitin-mediated interventions may need to be tailored to disease context. Finally, because these ubiquitin enzymes are also active in non-cardiac tissues such as neurons and skeletal muscle, systemic modulation could provoke unintended cross-organ effects. Addressing these complexities—through precise temporal control, cardiac-specific targeting, and human translational studies—will be key to determining whether ubiquitin-based manipulation of mitochondrial pathways can be safely and effectively leveraged against heart failure.

#### *Ubiquitination in cardiomyopathies and congenital heart disease*

Ubiquitin-dependent proteostasis is increasingly implicated in several forms of cardiomyopathy. For example, pathogenic variants and reduced expression of BAG3—a co-chaperone that cooperates with Hsp70 and E3 ligases—are established causes of genetic dilated cardiomyopathy (DCM); BAG3 loss impairs sarcomeric protein turnover and, more recently, has been shown to control TGF-β receptor 2 abundance in cardiac fibroblasts, linking BAG3 dysfunction to fibrosis in human DCM.^95^^,^^96^ Arrhythmogenic and desmosomal cardiomyopathies also intersect with ubiquitin-dependent pathways: abnormal turnover of desmosomal proteins (*e.g.*, desmoplakin, plakophilin) and altered ubiquitin–proteasome coupling have been reported in patient tissues and model systems, supporting a role for ubiquitin-mediated degradation in disease progression.^97^ By contrast, direct mechanistic evidence linking ubiquitin enzymes to many congenital structural defects is emerging but still limited: human genetic studies have identified the E3-regulator MIB1 (a Notch pathway E3 regulator) in bicuspid aortic valve and related left-sided lesions, with supporting mouse data, indicating that ubiquitin-linked regulators of developmental signaling can underlie certain congenital phenotypes.^98^

### Ubiquitination and arrhythmias: Regulating ion channel proteins

**Figure 7:**
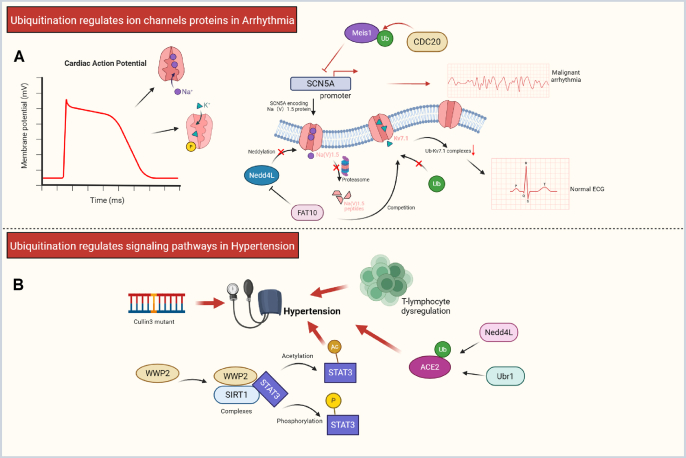
Roles of ubiquitination in regulating cardiac arrhythmia and hypertension. (**A)** Ubiquitination regulates ion channel proteins in cardiac arrhythmia. Normal cardiac rhythms rely on the coordinated function of cardiac ion channels. The protein Na(V)1.5 is encoded by the SCN5A gene. The E3 ubiquitin ligase CDC20 mediates ubiquitination of the transcription factor Meis1, which inhibits transcription of SCN5A and leads to arrhythmia. FAT10 binds to Kv7.1, preventing its ubiquitination and mitigating arrhythmia. **(B)** Ubiquitination regulates signaling pathways in hypertension. Mutations in Cullin 3 cause hypertension. The E3 ubiquitin ligases WWP2 and Nedd4L, and dysregulation of T lymphocytes can contribute to the development of hypertension. SCN5A, sodium voltage-gated channel alpha subunit 5; Nedd4L, NEDD4-like E3 ubiquitin protein ligase; CDC20, cell division cycle 20 homologue; FAT10, human HLA-F-adjacent transcript; Ub, ubiquitin; Cullin 3, subunits of the CUL3-RING (CRL3) ubiquitin ligase complex; ACE2, angiotensin converting enzyme 2; Ubr1, ubiquitin protein ligase E3 component N-recognin 1; Ac, acetylation; P, phosphorylation.

Arrhythmias represent a major form of cardiac dysfunction arising from abnormal electrical activity, with severe cases often resulting in sudden cardiac death.^99^ The maintenance of normal cardiac rhythm requires tightly coordinated activity of ion channels, including Na^+^, K^+^, and Ca^2+^ channels. Disturbances in their expression, trafficking, or degradation disrupt action potential generation and propagation, creating a substrate for malignant arrhythmias. Imbalances in these ion channels can result in severe arrhythmias.^100^ Growing evidence indicates that ubiquitination and ubiquitin-like modifications play pivotal roles in the regulation of ion channel homeostasis and arrhythmogenesis (Fig. 7A).

At the transcriptional level, ubiquitination can indirectly regulate ion channel expression. A representative example is the CDC20–Meis1–SCN5A axis. In mouse cardiomyocytes, the E3 ligase CDC20 mediates ubiquitin-dependent regulation of the transcription factor Meis1, thereby suppressing SCN5A transcription. Loss of SCN5A expression leads to dysregulation of the cardiac sodium channel NaV1.5, which in infarcted hearts predisposes to malignant ventricular arrhythmias.^101^ This pathway highlights how ubiquitination extends beyond protein degradation to influence the stability of transcriptional regulators critical for cardiac excitability.

At the post-translational level, ubiquitination directly controls the stability and surface expression of ion channels. The E3 ligase Nedd4L promotes ubiquitination and degradation of NaV1.5, reducing sodium current and impairing conduction. In hypertrophied hearts, Nedd4L activity has also been linked to reduced repolarizing potassium currents (*e.g.*, I–Kr), prolongation of the QT interval, and heightened arrhythmia susceptibility. Pharmacological or genetic inhibition of Nedd4L was shown to restore I–Kr and reduce arrhythmic events.^102^^,^^103^ These findings emphasize the chain-type and pathway-specific nature of ubiquitination, as different linkages (*e.g.*, K48, K63, or neddylation) may dictate distinct outcomes for channel trafficking, internalization, or degradation.

These findings emphasize the chain-type and pathway-specific nature of ubiquitination, as different linkages (*e.g.*, K48, K63, or neddylation) may dictate distinct outcomes for channel trafficking, internalization, or degradation.^104^ Beyond sodium channels, FAT10 also stabilizes the potassium channel subunit Kv7.1 by preventing its ubiquitination, enhancing I-Ks currents and contributing to improved repolarization stability.^105^ These observations suggest that targeting ubiquitin-like modifiers may allow selective protection of ion channel function without broadly suppressing ubiquitin-proteasome activity. In brief, ubiquitin pathways shape arrhythmogenesis by influencing channel gene expression, protein stability, and competitive modification, thereby creating dynamic heterogeneity in cardiac excitability. Several priorities should guide future research. First, mechanistic studies must link ubiquitination events directly to electrophysiological endpoints, using patient-derived cardiomyocytes or conditional genetic models rather than relying solely on protein abundance as a surrogate. Second, the functional consequences of specific ubiquitin linkages and ubiquitin-like modifications need to be systematically dissected to enable precision targeting of channel regulation. Finally, integrating human-induced pluripotent stem cell-derived cardiomyocytes, explanted cardiac tissue, and biomarker discovery will be essential for patient stratification and clinical translation. Only through this multidimensional approach can ubiquitin-based regulation of ion channels be transformed into viable antiarrhythmic strategies.

### Ubiquitination and hypertension: Regulating hypertension-related signaling pathways

As of 2025, hypertension continues to represent one of the most pressing global public health challenges, with both prevalence and hypertension-attributable mortality showing a persistent upward trajectory worldwide, underscoring the urgent need for mechanistic insight and new therapeutic strategies.^106^^,^^107^ Ubiquitin-dependent regulation has emerged as a convergent mechanism that links renal, vascular, and immune contributors to blood-pressure control (Fig. 7B). Below, we summarize representative findings, extract common mechanistic themes, and highlight the principal translational challenges.

**CUL3 and CRL3 biology—A nodal regulator.** Cullin-3 (CUL3), the scaffold of CUL3-RING ligases (CRL3), is genetically and functionally implicated in blood-pressure homeostasis. Mutations in CUL3 cause pseudohypoaldosteronism type II (PHAII) and familial hypertension, implicating defective substrate ubiquitination in disease pathogenesis.^108^ Conversely, restoration of CUL3 expression has been reported to exert vascular protective effects—at least in part by augmenting sonic-hedgehog (SHH) signaling, reducing endothelial apoptosis and oxidative stress, and improving endothelial function.^109^ Together, these observations position CRL3 activity as a nodal controller of vascular and possibly renal pathways relevant to hypertension.

**Vascular smooth muscle**—**WWP2 and STAT3-centered remodeling.** Ubiquitin ligases in VSMCs modulate phenotypic switching and contractile function. In angiotensin II (AngII)-driven models, the HECT-type E3 ligase WWP2 is up-regulated and interacts with the SIRT1–STAT3 complex. Mechanistically, WWP2 diminishes SIRT1's repressive effect on STAT3, thereby favoring STAT3 K685 acetylation and Y705 phosphorylation—modifications that promote pro-remodeling transcriptional programs in VSMCs. Genetic or pharmacologic suppression of WWP2 reduces hypertensive vascular pathology in preclinical models, highlighting a VSMC-intrinsic ubiquitin axis that mediates AngII responses.^110^

**Immune regulation and age-related hypertension.** Age-dependent alterations in T-cell function contribute to hypertension. The prostaglandin D2 (PGD2)–DP1 signaling axis in T cells modulates Th1 responses and, via enhancement of NEDD4L-mediated ubiquitination, promotes proteolytic turnover of the Th1 master regulator T-bet. This mechanism blunts age-related Th1 activation and mitigates AngII-induced hypertensive responses, suggesting that ubiquitin-mediated control of adaptive immunity is an important determinant of blood-pressure regulation in aging.^111^, ^112^, ^113^, ^114^

**ACE2 regulation and neurogenic contributions.** Angiotensin-converting enzyme 2 (ACE2) functions as a counter-regulator of the renin–angiotensin system; loss of ACE2 exacerbates vasoconstriction, sympathetic activation, and hypertension.^115^ Two E3 ligases—Ubr1 and Nedd4-2/Nedd4L—have been implicated in ACE2 ubiquitination and degradation in experimental hypertension, providing a mechanistic route by which ubiquitination can suppress ACE2 levels and thereby aggravate neurogenic or systemic hypertensive phenotypes.^116^^,^^117^

**Small-molecule modulation of ubiquitin signaling.** Targeting upstream signaling nodes to perturb ubiquitin-regulated pathways is an emerging approach. For example, the indole alkaloid tabunin (Tab) has been reported to interact with TAK1 and block AngII-induced TAK1 ubiquitination and phosphorylation, thereby inhibiting downstream NF-κB and JNK/p38 MAPK activation and attenuating hypertensive cardiac inflammation and remodeling in preclinical models.^118^ Such studies illustrate the feasibility of indirectly modulating ubiquitin-regulated inflammatory axes pharmacologically.

Collectively, ubiquitination influences hypertension by targeting diverse substrates—transcription factors, signaling kinases, and membrane proteins—with outcomes shaped by cell type, disease context, and modification linkage. Despite promising findings, challenges remain: pleiotropic effects across tissues, incomplete knowledge of linkage-specific mechanisms, and limited human validation. Progress will depend on cell-type–specific models, ubiquitinomics in patient tissues, and selective therapeutic strategies.

### Crosstalk between ubiquitination and other post-translational modifications

Interplay between ubiquitination and other post-translational modifications, such as phosphorylation, acetylation, SUMOylation, and O-GlcNAcylation, adds a crucial regulatory layer to cardiovascular proteostasis. These modifications frequently converge on the same residues or protein motifs, creating competition or cooperativity that dictates protein stability, localization, and function.^119^ Several representative mechanisms illustrate this complexity.

Phosphorylation-dependent ubiquitination (phosphodegrons) provides a direct link between kinase signaling and protein turnover: for instance, phosphorylation of target proteins can generate recognition motifs for E3 ligases, accelerating degradation. In mitochondrial quality control, PINK1-mediated phosphorylation of both ubiquitin and Parkin is required for Parkin activation and mitophagy, underscoring how phosphorylation licenses ubiquitin activity in stressed cardiomyocytes.^120^^,^^121^ Conversely, phosphorylation can also stabilize substrates by blocking ubiquitination, as seen in β-catenin signaling.^122^

SUMOylation–ubiquitination interplay has been observed in oxidative stress responses. Gao et al reported that in endothelial and cardiac cells, PKCδ phosphorylation enhances its SUMOylation, which in turn suppresses ubiquitination and proteasomal clearance, shaping cell fate under stress.^123^ In parallel, SUMO-targeted ubiquitin ligases (STUbLs) can convert SUMOylated substrates into ubiquitination targets, linking SUMO modification to protein turnover in vascular cells.^124^

Acetylation and ubiquitination also show extensive cross-regulation. Lysine acetylation often competes directly with ubiquitination at the same residues. In cardiomyocytes, histone deacetylases control transcription factor acetylation (*e.g.*, NF-κB, MEF2), thereby altering their ubiquitin-dependent stability and transcriptional activity.^125^ Pharmacological inhibition of HDAC6 increases tubulin acetylation, enhances autophagy, and cooperates with the ubiquitin–proteasome system to clear misfolded proteins, protecting against proteotoxic cardiomyopathies.^126^

Metabolic modifications such as O-GlcNAcylation further influence ubiquitin signaling. O-GlcNAcylation of transcription factors and kinases can alter their recognition by E3 ligases, thereby linking hyperglycemia and metabolic stress to maladaptive ubiquitin signaling in diabetic cardiomyopathy.^127^ Similarly, redox modifications of cysteines in ubiquitin enzymes affect E3/DUB activity under ischemia–reperfusion conditions.^128^

Together, these examples emphasize that ubiquitin pathways rarely act in isolation. Crosstalk with phosphorylation, acetylation, SUMOylation, and metabolic post-translational modifications transforms transient signaling into stable proteostatic changes that drive processes such as hypertrophy, fibrosis, inflammation, mitophagy, and vascular remodeling. From a translational perspective, this complexity cautions against targeting ubiquitin ligases or DUBs in isolation; instead, therapeutic strategies that exploit post-translational modification interfaces (*e.g.*, kinase–E3 axes, acetylation–ubiquitination switches, or SUMO–ubiquitin coupling) may achieve greater specificity.

## Conclusions

Ubiquitination is a fundamental post-translational regulator of cardiac and vascular biology, orchestrating protein turnover, signal transduction, organellar quality control, and stress responses that together shape cardiomyocyte function, fibroblast activation, vascular remodeling, and inflammation. Our review synthesizes recent advances linking specific E3 ligases, DUBs, and ubiquitin-like modifiers to major cardiovascular pathologies (Table 1), and highlights both mechanistic opportunities (substrate-biased modulation, post-translational modification crosstalk) and translational challenges (enzyme redundancy, systemic pleiotropy, cardiac safety). Early clinical experience with broad ubiquitin–proteasome system inhibition has exposed important safety limits,^125^^,^^126^ while selective strategies (DUB/E3 modulators, targeted degraders) provide feasible translational routes but require tissue selectivity and rigorous cardiac safety assessment.^129^ Taken together, the field is poised to translate mechanistic insights into targeted therapies—provided that future work addresses specificity, spatiotemporal control, and human validation.

**Table 1:** E3 ubiquitin ligases and DUBs functions and regulation.

Enzymes	Type	Targets	Mechanisms	Biological response	Reference
TRIM72 (MG53)	RING	NF-κB pathway	Reduces apoptosis and oxidative stress in the aged heart	Alleviates heart failure	^133^
TRIM38	RING	TAK1/MAPK axis	Interacts with and degrades TAB2 and TAB3, inhibits TAK1 phosphorylation, and negatively regulates MAPK signaling, reducing fibroblast proliferation and secretion	Alleviates cardiac fibrosis	^134^
TRIM44	RING	TLR4/NOX4 axis	Interacts with TLR4, increases NOX4 expression, and increases ferritin deposition levels	Aggravates cardiac hypertrophy	^135^
		TAK1/MAPK axis	Inhibiting k48-linked polyubiquitination maintains TAK1 stability, phosphorylates increased TAK1 expression, and activates MAPKs	Aggravates cardiac fibrosis	^75^
TRIM27	RING	P53	Interacts with and promotes ubiquitination of P53 and inhibits apoptosis and inflammatory responses	Mitigates myocardial ischemia–reperfusion injury	^136^
TRIM64	RING	IκBα/NF-κB pathway	Promotes ubiquitination of IκBα, activates the NF-κB pathway, and promotes ox-LDL-induced THP-1 macrophage foam cell formation	Aggravates atherosclerosis	^44^
TRIM7	RING	c-Jun/AP-1 pathway	Activation of the c-Jun/AP-1 signaling pathway promotes VSMC proliferation and migration	Aggravates atherosclerosis	^137^
TRIM37	RING	TRAF2/NF-κB pathway	Negative regulation of TRAF2 ubiquitination in foam cells mediates NF-κB translocation to the nucleus and exacerbates ox-LDL-induced foam cell formation	Aggravates atherosclerosis	^46^
TRIM24	RING	Dysbindin/SRF pathway	Protects dysbindin from TRIM32 degradation and activates Rho-dependent serum response factor (SRF) signaling	Aggravate cardiac hypertrophy	^67^
TRIM32	RING	Dysbindin/SRF pathway	Degradation of dysbindin in neonatal rat ventricular cardiomyocytes attenuates Rho-dependent activation of serum response factor (SRF) signaling	Alleviates cardiac hypertrophy	
		XIAP factors	Inhibits the X chain of apoptosis inhibitors	Promotes cardiomyocyte apoptosis	
		P53, Caspase-3/-7	Activates P53 and caspase-3/-7	Promotes cardiomyocyte apoptosis	
TRIM14	RING	AKT pathway	Activates the AKT pathway	Aggravates cardiac hypertrophy	^66^
		NF-κB pathway	Enhances endothelial activation by activation of the NF-κB signaling pathway	Aggravates atherosclerosis	^138^
TRIM65	RING	Jak1/Stat1 pathway	Inhibits mitochondria-dependent apoptosis and autophagy via the Jak1/Stat1	Attenuates ISO-induced cardiac hypertrophy	^89^
TRIM63 (MuRF1)	RING	CnA/NFAT axis	Increases ubiquitination and decreases protein stability of CnA, and decreases nuclear factor of T cells (NFAT) activity	Alleviates cardiac fibrosis	^55^
TRIM10	RING	PTEN/AKT pathway	Promotes PTEN ubiquitination and activates the AKT signaling pathway	Aggravates myocardial hypertrophy	^139^
TRIM35	RING	PKM2/GATA4/6 axis	Increased ubiquitination of nuclear PKM2 promotes its degradation, decreases GATA4/6 stability, and increases P53 in cardiomyocytes	Promotes heart failure	^140^
MDM2	HECT	RXRβ	RXRβ polyubiquitination degradation leads to mitochondrial damage and endothelial pro-inflammatory factor production	Aggravates atherosclerosis	^41^
		HIF1α/HIF2α	Dynamic regulation of HIF1α/HIF2α protein stability leads to HIF imbalance and reduced pro-angiogenesis	Myocardial capillary angiogenesis disorders	^69^
WWP2	HECT subtypes NEDD4	Septin4 protein	Promotes Septin4 ubiquitination and degradation, thereby inhibiting Septin4-PARP1 endothelial damage complex formation	Alleviates oxidative stress-associated atherosclerotic radical hypertension	^40^
		SMAD2 pathway	Interacts with and promotes the transcriptional activity of SMAD2	Aggravates TGF-β1-induced pathological cardiac fibrosis	^74^
		SIRT1–STAT3	WWP2 forms a complex with SIRT1-STAT3 in vascular smooth muscle, which in turn reduces the inhibitory effect of SIRT1 on STAT3	Hypertension vascular disease	^110^
NEDD4L	HECT subtypes NEDD4	YAP/NEDD4L/ACS4L	YAP promotes NEDD4L transcription and ubiquitination and degradation of ACSL4, and inhibits cardiomyocyte iron metabolism	Mitigates myocardial ischemia–reperfusion injury	^59^
		Nav1.5	Ubiquitinates and degrades the Nav1.5 channel	Arrhythmia	^141^
		ACE2	Promotes ACE2 ubiquitination	Neurogenic hypertension	^117^
RNF207	RING	TAB1 protein	Promotes ubiquitination and destabilization of TAB1 and activates downstream p38 and c-Jun N-terminal kinase (JNK)1/2 signaling pathways	Aggravates myocardial hypertrophy	^64^
RNF13	RING	p62-NRF2/HO-1	Interacts with p62 and promotes downstream NRF2/HO-1 signaling activation	Alleviates cardiac hypertrophy	^65^
USP11	DUBs	CTRP9/USP11/CD36 axis	CTRP9 decreases USP11 levels, which in turn decreases CD36 levels, and inhibits the accumulation of intracellular lipids and cholesterol, preventing the transformation of macrophages into foam cells	Alleviates atherosclerosis	^47^
		TRAF3	Deubiquitination of TRAF3 induced cardiomyocyte injury	Aggravates myocardial ischemia–reperfusion injury	^56^
USP10		Sirt6–AKT pathway	Increases ubiquitination of Sirt6 and down-regulation of the Akt signaling pathway	Alleviates cardiac hypertrophy	^142^
USP18		TGFβ1-p38/c-Jun1/2 pathway	Blocks the transforming growth factor-beta-activated kinase 1-p38/c-Jun N-terminal kinase 1/2 signaling cascade	Inhibits cardiac hypertrophy and delays cardiac dysfunction	^143^
USP19		TAK1/p38/JNK1/2 pathway	Inhibits TAK1-p38/JNK1/2 transactivation	Aggravates myocardial hypertrophy	^144^
USP25		SERCA2a protein	Deubiquitination maintains the stability of the SERCA2a protein and maintains normal calcium ion endocytosis channel function	Alleviates cardiac hypertrophy and cardiac insufficiency	^70^
USP2		Not found	Inhibits inflammatory response, inhibits fibrosis, and attenuates oxidative stress	Relieves cardiac remodeling	^145^
USP4		TAK1–JNK1/2/P38 axis	Overexpression of USP4 attenuates activation of TAK1-JNK1/2/P38 signaling in response to hypertrophic stress	Alleviates cardiac hypertrophy	^146^
USP22		SIRT1/p53/SLC7A11	Down-regulation of SIRT1 and SLC7A11 and up-regulation of P53 attenuate iron metamorphosis-induced cell death	Mitigates myocardial ischemia–reperfusion injury	^61^
USP28		PPARα–Mfn2 axis	Deubiquitinates and stabilizes PPARα, promotes Mfn2 (mitomycin 2) transcription, and inhibits mitochondrial morphology defects	Alleviates diabetic cardiomyopathy	^71^
UCHL1		CD36	UCHL1 deletion promotes CD36 protein degradation to reduce foam cell formation	Alleviates atherosclerosis	^48^
ITCH	HECT	Wnt/β-catenin pathway	Ubiquitination degrades Dvl protein and inhibits the Wnt/β-catenin signaling pathway	Attenuates cardiomyocyte hypertrophy	^68^
Pellino1	HECT	NF-κB/TGF-β pathway	Knockdown of Pellino1 prevents binding of NF-kB and AP-1 to the TGF-β1 promoter region and inhibits transcriptional activation of TGF-β1	Mitigates cardiac fibrosis	^73^
Atrogin-1 (Fbxo32)	Cullin-RING	FoxO1/3	Enhances FoxO1/3 activity by overexpression of Atrogin-1	Alleviates aging-related cardiac fibrosis	^147^
FAT10	Ubiquitin-like protein	Nav1.5/Nedd4L	Reduces binding of Nav1.5 to the ubiquitin E3 ligase Nedd4L protein	Prevents ischemia-induced ventricular arrhythmias	^104^

## Therapeutic translation of ubiquitin pathway insights

The mechanistic insights reviewed above highlight ubiquitination as a central regulator of cardiomyocyte homeostasis, fibroblast activation, vascular remodeling, and immune responses. However, therapeutic translation requires consideration of two critical dimensions: crosstalk among ubiquitin-related pathways and the potential for off-target consequences.

Early clinical experience underscores both the promise and the pitfalls of targeting the ubiquitin–proteasome system. Broad proteasome inhibitors, such as bortezomib and carfilzomib, are effective in oncology but have produced clinically significant cardiotoxicity, limiting their applicability for chronic cardiovascular disease.^129^^,^^130^ In contrast, more selective interventions are emerging. For example, deubiquitinase USP25 stabilizes SERCA2a and attenuates pathological hypertrophy in animal models, highlighting how targeted stabilization of protective proteins could be cardioprotective.^70^^,^^131^ E3 ligase modulators and inhibitors (*e.g.*, MDM2 inhibitors) are progressing in oncology trials,^132^ and targeted protein degradation platforms such as PROTACs and molecular glues demonstrate that substrate-specific modulation is feasible in humans.^129^ These advances suggest that cardiovascular applications are technically achievable, but require tissue-specific delivery and careful safety profiling.

The therapeutic promise of these strategies lies in their capacity for substrate- and context-specific modulation, potentially leveraging kinase–E3 axes, acetylation–ubiquitination switches, or SUMO–ubiquitin coupling to refine selectivity. Yet redundancy across E3s and DUBs, together with systemic functions of shared substrates, remains a barrier. Moving forward, progress will depend on integrating mechanistic insight with biomarker-guided design, combining ubiquitin-focused proteomics with phospho- and acetylomics, and establishing early cardiac safety endpoints in clinical trials. Such a network-aware approach will help harness the plasticity of ubiquitin pathways while minimizing the risk of destabilizing cardiovascular homeostasis.

### Future perspectives

Future research should focus on mapping spatiotemporal ubiquitin landscapes with cell-resolved proteomics, defining ligase–substrate specificity in structural and contextual detail, and developing ubiquitin-based biomarkers to guide patient stratification. Equally important is advancing tissue-targeted and substrate-biased therapeutics such as PROTACs and molecular glues with embedded cardiac safety endpoints, while prioritizing human validation through genetics, patient tissues, and clinical cohorts. Collectively, these priorities call for a network-aware research program that integrates advanced proteomics, mechanistic biology, structural insight, and clinical phenotyping, thereby clarifying fundamental principles of ubiquitin regulation in cardiovascular disease and paving the way toward safer, more specific ubiquitin-directed therapies.

## Rights and permissions

This article is licensed under a Creative Commons Attribution 4.0 International License, which permits use, sharing, adaptation, distribution, and reproduction in any medium or format, as long as you give appropriate credit to the original author(s) and the source, provide a link to the Creative Commons licence, and indicate if changes were made. The images or other third-party material in this article are included in the article's Creative Commons licence, unless indicated otherwise in a credit line to the material. If material is not included in the article's Creative Commons licence and your intended use is not permitted by statutory regulation or exceeds the permitted use, you will need to obtain permission directly from the copyright holder. To view a copy of this license, visit http://creativecommons.org/licenses/by/4.0/.

## CRediT authorship contribution statement

**Jingjing Zhu:** Writing – original draft. **Zhimei Qiu:** Writing – original draft. **Yi Xu:** Writing – original draft. **Qing Guo:** Writing – original draft. **Shuangya Yang:** Supervision. **Yongchao Zhao:** Writing – review & editing. **Bei Shi:** Visualization, Funding acquisition.

## Funding

This work was supported by a scientific research project from the 10.13039/501100001809National Natural Science Foundation of China (No. 82470291, 82460055).

## Conflict of interests

The authors declared no competing interests.
